# Dr. Hutchinson's Dissertation on Infanticide

**Published:** 1820-12-01

**Authors:** 


*A Dissertation on Infanticide, in its Relations to Physiology and Jurisprudence.* By William Hutchinson, M. D. F. L. S. Octavo, pp. 99. London, 1820.

It is curious to observe the difference which a few parallels of longitude make in our moral sentiments, religious feelings, and legal obligations. We have seen the crime, which occupies the work before us, perpetrated without the least compunctious visitings of conscience, or danger of jurisprudential retribution. Parents every day commit their offspring alive to the rivers or streets of China, with as little ceremony as we would a litter of kittens or a useless puppy. Such are the effects of a population arrived at its ultimatum. We know not but this may yet be the state of England. In the mean time, Nature still cries out against infanticide here, and the law inflicts a severe penalty on the commission of it. Our readers are all aware that, till very lately, medical jurisprudence was greatly neglected in this country. The example of our continental brethren, however, has now roused the attention of English writers, and, to our knowledge, five authors are, at this moment, preparing for the press, massive volumes of translations, compilations, abridgements, and analyses of German and other continental works on legal medicine. Dr. Hutchinson, however, will probably anticipate them all. The literary appetite of this gentleman might well compete with that of Domine Sampson; and his literary lucubrations are truly "prodigious." In his late controversy with Dr. Wilson Philip, he states that the proemium to the 43rd volume of the Medical and Physical Journal (of which periodical work Dr. Hutchinson avows himself, in the book before us, to be the conductor) was written in a fortnight, while, of course, he had the usual labour of editing the journal on his hands besides. Now as this proemium professes to lay before us the semi-annual progress of medicine and its auxiliary branches, in Europe, America, and great part of the civilized globe, containing reviews of, quotations from, or references to, several hundred volumes, (many of which were supposed never to have seen this country,) we do again assert, that Dr. Hutchinson's literary powers are not only "prodigions," but, that they appear to us almost super-human, and, in a darker age, would certainly have afforded suspicion that the author had assistance from the black art. It excited surprize that Dr. Johnson should have written his Rasselas in ten days. But what was this tale of fancy, compared to the learned and accurate proemium of Dr. Hutchinson?—a sonnet to a cyclopœdia.—Far be it from us to admit the sarcastic insinuation thrown out by Dr. Philip—that the reading of a work, and the reviewing thereof, have no necessary connexion in some systems of things. No, no! This would be admitting a doctrine that saps the foundation of our own calling. We solemnly affirm, then, that *all* reviewers peruse carefully *all* works, and are intimately acquainted with *all* subjects, practical, theoretical, physical, and metaphysical. We moreover declare, in the name and behalf of the whole critical brotherhood, that *no* work is ever slurred over, or carelessly quoted—that *no* perversion of an author's sentiments is ever exhibited in a review*—that *no* puffs are ever inserted in the shape of criticisms—that *no* secret influence could induce a reviewer to laud a Charlatan, and vituperate a Cooper—that *no* reviewer is under the slightest control of a publisher or proprietor, but, on the contrary, is the sole arbiter of his own actions—that *no* reviewer did ever openly advocate the cause of a quack, or wilfully caricature the experiments of a Philip and a Brodie—that *no* reviewer did ever crouch before a tyrant master, or renowned infidel knight, and publish in a medical journal, the raving rhapsodies about "moon and motion" engendered in the chaotic brain of a radical Sherif—that *no* reviewer did ever admit the assistance of *auto-criticism* from certain modest authors; although such a proceeding would be perfectly justifiable, since who can pretend to so

* And here we cannot help remonstrating most angrily with Dr. Halloran of Cork, on account of the following passage, which he lately wrote to a friend of his in London, and which appears to be in unison with the Worcester—shire *Philipics*.

"I have passed muster (says Dr. Halloran, speaking of his work on Insanity) as far as I know, tolerably well with the reviewers, except with the *Medical and Physical Journal;* in which, a critique appeared, in my mind, the most unwarrantable and inconsistent I ever met with. In that critique, I am dragged before the public as having entirely failed in a department of medical science on which *I had not entered,* and which, I expressed my determination to avoid. In having maintained this resolution throughout, I am taxed with gross ignorance on *all* subjects connected with pathology, and am set down as—a friend to *materialism!* Shame on such reviewers! I did not hesitate to communicate my sentiments to the author of this rediculous tirade, given, as is obvious, with no better view, than as a prelude to those favourite sentiments of which the individual seems not a little vain. He appears somewhat fretted by my observations; and so he well might, though he deserved much more. I do not know his name, nor do I care to do so. He wrote to me in reply, but did not append his signature."

Lest Dr. Halloran should accuse *us too* of misrepresenting his sentiments, we have kept an attested copy of the passage, though we are quite sure that Dr. H. is all in the wrong, for a reviewer *cannot* err.

intimate an acquaintance with the contents of a work, as the man who wrote it?—that *no* reviewer did ever place a book for review in the hands of the professed enemy or rival of its author; although such a step would also be perfectly lawful, and even desirable, since it would insure a *long* critique without any expence, and without the least risk of fulsome adulation—finally, that *all* these things are strictly true, "without, (as Dr. Harrington would say,) one single anomaly."

But to return from this digression in defence of critics. We think that Dr. Hutchinson's compilation, (which we take to be a more proper title than "dissertation") is a useful, or at least a respectable little work, its greatest fault being one that seems inseparable from every thing connected with *law* —namely, *verbiage*. Neither can its English dress conceal its foreign origin, as some *minutiæ* will presently evince. We shall endeavour therefore, chiefly for the benefit of those who may not have an opportunity of furnishing themselves with the work itself, to draw up a concise analysis of its most prominent and useful features, disentangling, as far as we can, the ideas from the load of words with which they are frequently oppressed and obscured.

Infanticide, according to law, is the destruction of a child's life, either during its birth, or within a few days after it is born. We shall pass over the first five sections of the work, containing common-place directions respecting our conduct in proceeding to examine the body of a child, supposed to be murdered. In the sixth section the anatomical examination of the body is discussed; and here we are somewhat *Hibernically* directed, in noticing "the *external* appearances," and, before any dissection, to first examine whether the child has had the misfortune to be born without a head, brain, *lungs*, *heart*, or *alimentary canal*. P. 7. An acephalous infant might certainly be easily recognized, without dissection; but how a surgeon is to ascertain that a child has, or has not a *heart* or *intestines*, "before he begins to dissect any part of it," this deponent knoweth not.

Here our author makes a long digression on the progressive developement of the fœtus, *ab ovo usque ad partum*. We shall only be able to present a few specimens of the *valuable and minute* observations of our foreign medico-legal physiologists. We shall begin at a *comparatively* advanced period of man's rise in this world—namely at the 30th day of utero-gestation, when he is as large as an *ant*, according to Aristotle—as a *barley-corn*, according to Burton—as a *fly*, according to others—as the *malleus* of the *tympanum*, according to Baudelocque. P. 7.—At 45 days, the length of the fœtus is about ten lines—at 60 it is two inches long—at the end of the 5th month the mother quickens, and at that period it is important to know that—"the scrotum is of a bright red (not blue) colour." But when we had read nearly six pages of this minute description, we were warned, that after all, the structure of the soft parts would not afford any positive evidence, "because diversities in this respect, will exist from varieties in the age and constitution of the mother, and from other circumstances affecting especially the fœtus itself." 12. Here then, as in most other cases of German minuteness, we have rolled the stone up to the top of the hill, only to have the mortification to see it roll down again, with much more velocity.

M. Beclard appears to have arrived at more satisfactory conclusions respecting the age of the embryo, by osteological researches.

"After two months have elapsed from the period of conception, the skeleton is about 4 inches and 3 lines in length; that of the spine being 2 inches. At three months, the former is 6 inches, and the proportion of the spine as $2\frac{2}{3}$ to 6. At 4 months and a half, it is 9 inches, and the spine 4; at six months, 12 inches, the spine 5; at $7\frac{1}{2}$ months, 15 inches, the spine $6\frac{1}{3}$; at nine months, or the period of birth, it is ordinarily from 16 to 20 inches in length, or, at a medium, 18; and the spine is in the proportion of $7\frac{1}{4}$ to 18 to the whole length of the body. These calculations were made from observations on about fifty fœtuses, at each of the periods above indicated." 13.

Dr. Hutchinson cites numerous authorities respecting the *weight* of the fœtus, at the full term of utero-gestation; but all is discordance here too. An average weight may probably be stated at six or seven pounds. The *length* of the fœtus at the full term "is ordinarily from nineteen to twenty-two inches."

The external marks of violence are to be carefully examined. We know that superficial livid marks, on the under surfaces of dead bodies, arising from stagnation of the blood in the small vessels of the skin, may be regarded by the ignorant as signs of violence; but a layer of the skin being removed, shews that the lividity is confined to that organ solely.

Infants are most commonly destroyed by wounds, contusions and strangulation.

*Wounds*. It is always important to ascertain whether these have been made during life, or after death. In the *former* case they present red or bloody surfaces, with ecchymosis; and when death has not instantly followed their infliction, their edges will be somewhat tumid and retracted, the surrounding skin being of a reddish hue. When several days have elapsed, they will, of course, present symptoms of the suppurative process.

When the wounds have been inflicted on the body after the circulation of the blood has ceased, and the body has become cold, the blood being coagulated in the vessels, their surfaces will be pale, and without tumefaction, retraction, or surrounding ecchymosis. These characters, however, will not be near so distinct when the wounds have been inflicted soon after death; the body being yet warm, the blood fluid, and the muscles retaining some degree of contractility.

*Contusions.* These, when effected during life, are always accompanied by more or less ecchymosis. When this is superficial, and the subject outlives its production, the part presents a red or bluish spot, soon assuming a deep livid or leaden hue, changing afterwards to a yellow, and requiring seven or eight days for its ultimate disappearance.

*Post mortem* bruises are of a deep, or purplish brown colour, attended with no diffused infiltration of blood in the cellular tissue, or elevation of the skin. Here, however, as in almost every part of infanticide, the " glorious uncertainty of the law," is eclipsed by the yet more glorious uncertainty of legal medicine, and every conclusion ends in—nothing being concluded.

" It should be borne in mind that the child may suffer such a degree of pressure, and other violence, during parturition, that there will be found in different parts of its body, after its birth, *ecchymoses, tumefactions, fractures of the bones, and laxations of the joints.*" 24.

This legal medicine will afford excellent materials for the bench. When it comes to be studied by *lawyers,* there will be an end to conviction of crime, for it will be absolutely impossible to prove any one thing, as far as the medical evidence is concerned!

*Suffocation.* The external indications of this are redness or lividness, with tumefaction of the face, prominence and redness of the eyes, projection of the tongue from the mouth, and a frothy oozing of mucus from the same. All these, however, may be produced " by the viscid mucus, naturally existing about the pharynx and glottis in newly-born infants, getting into the trachea, especially if the infant has lain on its back for some time after its delivery." 27. As for a livid circle round the neck, which might induce a common spectator to suspect the operation of a ligature, the medico-legal investigator will shew that this is " capable of being produced by rigid and forcible contraction of the orifice of the uterus, or from the navel-string being twisted round the neck of the infant, which may have suffocated it." 28. We do not see that an internal examination removes this source of uncertainty; for if the funis may strangle the infant, without flattening the trachea or larynx, so may a garter or handkerchief applied with a similar degree of tightness. In short, we are here, as usual, left in doubt and uncertainty.

On the subject of the unbilical cord, nothing is decided but that every thing is uncertain. The child may or may not bleed to death, if the cord be cut and not tied; or if it be left untied, and in continuity with the placenta. A learned list of German disputants, to the number of fourteen or fifteen, with the titles of all their disputations, is given at page 30 of our author's work. These worthies had a tremendous paper war, towards the close of the seventeenth century, "respecting the necessity of tying the cord to prevent hæmorrhage." Peace to their tomes! They are not likely to be ever opened in this country but by Dr. Hutchinson himself.

The signs of a child having died from umbilical hæmorrhage are stated to be, a bluish or dull pallid colour of the whole surface of the body, paleness of the viscera, want of blood in the large vessels, especially the veins, and in the cavities of the heart.

"But such effects cannot be attributed to hæmorrhage from the navel-string, except when no other means for its occurrence are present, and when the body of the infant appears to be well constituted, with a full and free development of the cord. When this part is shrivelled, in a body recently born, and its vessels in a state of extreme collapse, it may be supposed that the infant wanted blood for some time before its birth.

"Should there be evidence of hæmorrhage from the umbilical arteries or their ramifications, it must be borne in mind, that it might have taken place from the placenta during labour; or, as Mr. John Burns states, rupture of the vessels of the cord may have happened at that time, and fatal hæmorrhage thence resulted." 32.

This leaves us, of course, in the region of conjecture, where we began.

Into the long and minute directions for opening the head, spinal canal, and thorax, we shall not enter, as they are familiar to every one who knows the anatomy of the body, and to others they are unintelligible. The thorax, however, leads to a host of physiological digressions and discussions relative to "the peculiarities of the fœtal circulation—the changes effected in the lungs and their blood vessels by respiration—the theory of the several tests to which the lungs have been exposed in relation to the subject of infanticide—and the appearances produced in the same organs by drowning and respiration of deleterious gases." In wading through the contradictory and unsatisfactory statements and tests respecting the lungs of infants, and the fact of respiration having been commenced or not, we did curse the hour when first we undertook the task of reviewing, and also the man who first invented forensic medicine, to puzzle the brains of judge, jury, counsellor, and doctor! After labouring for some hours to come to something like a conclusion, the following statement plunged us in despair, and very nearly induced us to plunge book and manuscript into the fire.

"Lecieux relates the results of four hundred examinations of bodies of children, made at the Hospice de la Maternité, at Paris, for the purpose of furnishing some evidence on this subject; and the results of them are almost as various as it was possible for them to have been, within a certain range." 55.

Although theories are seldom very satisfactory to any but those who invent them, yet the theory of drowning, at page 64 of Dr. Hutchinson's work, carried complete conviction to our minds the moment we read it. It is beautifully simple; namely, that the cause of death, in drowning, "essentially depends, in the generality of cases, on the *want of air.*"

It is not to be expected that the signs of death from drowning are exempted from the cloud of ambiguity which wraps every medico-legal subject in cimmerian darkness. In short, there is not one single *decisive* criterion of a child's having been drowned unless there be present in the water found in the lungs, "pieces of straw, weeds, insects, or other foreign substances similar to what are found in the water when the body was discovered." 65.

Death from the vapour of charcoal is attended, it is said, with several peculiar appearances. The body preserves its heat for a long time after apparent death—there is great accumulation of black and very fluid blood in the veins, and hardly any in the arteries—the vessels of the lungs and brain are especially gorged with this fluid—the face is red, and somewhat tumefied—the eyes are bright, and the lips have a vermilion hue.

Our limits forbid us pursuing the analysis of this little work any farther; nor do we mean to offer any opinion on the merits of the compilation itself. This much we may venture to say, that if every part of medical jurisprudence be handled so minutely as the subject of infanticide, the whole range of our hitherto professional studies will be a mere cipher compared with that of forensic medicine! And after all, we venture to predict that this medico-legal cyclopœdia will furnish the means of tenfold more embarrasment to the medical practitioner, when cited before the tribunals of justice, than he before experienced. We conscientiously declare that, after perusing carefully the treatise on infanticide before us, we should not be able to give one single iota of positive evidence, in a court of law, on the subject which we have been studying, and as derived from anatomical and physiological evidence alone. How far moral and circumstantial evidence might enable us to make up our minds, we cannot say; but without this, all appears to be involved in doubt, and subject to deception. As soon as these medico-legal dissertations get into the hands of lawyers, medical men *must* study the subjects in their own defence—and then God give them power and patience to store their memories with such a load of physical subtleties, together with strength of nerve and quickness of intellect to meet the quibbles and cross-questions of the long robe gentry, furnished as they will then be with ample materials to "make the worse appear the better cause."

We cannot close this little volume without again bearing testimony to Dr. Hutchinson's high literary powers and unwearied industry. As an able cotemporary rival we admire his talents for minute research, and we hesitate not to publicly express that admiration. May he respect himself, and never lose sight of integrity, candour, and impartiality, which ought to characterize a medical author, and are not less necessary than talents in the conductor of a public journal!

